# Department of Therapeutics

**Published:** 1895-01

**Authors:** David Cerna

**Affiliations:** Demonstrator of Physiology and Lecturer on the History of Medicine in the Medical Department of the University of Texas, Etc.; Galveston


### Current Medical Literature.

### DEPARTMENT OF THERAPEUTICS.

UNDER THE CHARGE OF DAVID CERNA, M. D., PH. D.,
Demonstrator of Physiology and Lecturer on the History of Medicine in the Medical Department of the University of Texas, etc.

QUININE IN THE TREATMENT OF CHOLERA.—In an interesting paper, Erskine B. Fullerton, of Columbus, Ohio (*N. Y. Medical Journal*, August 18, 1894), draws the following conclusions: Ten grains of quinine in powder, diffused through a small quantity of water, or in acid solution, at hourly intervals, until twenty to forty grains have been given, afterward *pro re nata*, should be the ordinary instructions; the same dose at half-hourly intervals for a sufficient time in collapsed or in *foudroyant* cases; smaller doses, perhaps, at longer intervals in choleraic diarrhœa. There should certainly be retained, of other treatment, appliances for the restoration of heat; saline hypodermoclyses to supply lacking serum to the blood; morphine hypodermics to allay pain and cramps, with enteroclyses of quinine where, as past experience shows rarely to have been the case, the remedy is vomited; and in the sequent enteritis or otherwise persistent diarrhœa, calomel in small doses should not be lost sight of. That by so treating our patients we may hope for a mortality in collapsed and collapsing cases of about 14 to 25 per cent. only; that by earlier administration of the remedy, instead of the use of other agents that have heretofore permitted so many cases to run on into collapse and death, we may reduce the mortality in such cases to 2 to 5 per cent. only, seems a fair assumption for the best of reasons—i. e., it should be so, and so far it always has been so.

THE BEST TREATMENT IN HEART DISEASE.—Robert H. Babcock, of Chicago, Ill. (*The Journal of the American Medical Association*, November 17, 1894), concludes an able paper on the above subject as follows: 1. The position taken by Fraentzel, that rest is injurious in the treatment of all forms of heart disease is untenable, and the reasons he assigns are incorrect. Prolonged rest is detrimental, undoubtedly, in cases of enlargement of the heart without valvular disease, particularly if secondary to arterio-sclerosis, and in cases of fatty or other degeneration of the cardiac muscle. 2. The cause, however, lies in the circulation outside of the heart and not, as stated by Fraentzel, in the liability of cardiac, like striped voluntary muscle, to degenerate as a result of prolonged inaction, since the heart muscle can not during life be subjected to complete repose. 3. When compensation has become destroyed in valvular lesions of the heart, particularly mitral stenosis and aortic incompetence, rest is indicated theoretically and is beneficial in practice. 4. Bradycardia would theoretically contra-indicate prolonged rest. 5. On the other hand, it is called for in paroxysmal tachycardia, but should not be maintained, after having shown its powerlessness to affect the heart rate. 6. Acute inflammatory or degenerative affections of the heart indicate rigid rest in the recumbent position.

---

THE USE OF ANTIPYRIN IN LARGE DOSES.—One case of hysterical fits and two cases of chorea are reported by McCall Anderson (*British Medical Journal*, December 1, 1894), to show the good effects of antipyrin administered in large doses. According to the writer, antipyrin is the most valuable of all the coal-tar derivatives. The initial dose should not exceed ten or, at most, fifteen grains. When this quantity is well borne it should be increased cautiously to forty and even fifty grains three times a day. The author concludes that: 1. Antipyrin is not the dangerous drug which some observers have led us to suppose. 2. It may be given in safety in large doses, even in the case of children, in most cases, although the initial dose must be small, and it must be slowly and cautiously increased, the patient being always carefully supervised. 3. In large doses it often yields surprisingly good results, and in chorea it is the only medicine from which cures may confidently be anticipated.

---

ACTION AND USES OF STRONTIUM SALICYLATE.—In a study of the action and therapeutic uses of strontium salicylate, H. C.

Wood, of Philadelphia, Pa. (*University Medical Magazine*, January, 1895), gives the results of his experience. He has employed the salt in a large number of cases, in doses of from 15 to 120 grains a day. The general result of these trials shows that in doses of from 5 to 10 grains, given after meals, the salt very commonly improves digestion, and in the dose of 5 grains an hour after meals, in flatulent dyspepsia and various conditions of tendency to fermentative changes in the alimentary canal, it is a useful intestinal antiseptic, which has seemed to the author to give better results than do salol, naphthol, or other of the older antiseptic remedies. It does not produce cinchonism as readily as do the older salicylates, but it is entirely capable of causing a pronounced degree of cinchonism. He has not been able to test the drug in acute articular rheumatism, but it would probably be less efficacious than the ammonium salicylate. In muscular or subacute rheumatism as well as in chronic gouty conditions with a tendency to digestive disturbance, he has found it to be a very valuable remedy, exerting the action of the salicylate upon the diathesis, and improving instead of injuring the digestion. It may be given in solution, but is best administered in capsules; a five-grain capsule is of moderate side, and of these two or more may be taken at once. It is probable that it would be well administered in compressed tablets, but in that way the observer has not tested it. The taste of strontium salicylate is similar to but distinctly less offensive than that of the ordinary salicylates, so that if preferred it may be given in weak solution.

---

THE SERUM TREATMENT OF DIPHTHERIA.—In a discussion at the Munich Bezirksverein (*Munchener Medicinisehn Wochenschrift*, November 6, 1894), Buchner observed that this serum, experimented with in various laboratories, has yielded excellent results, yet, to prevent disappointment, caution is needed in drawing conclusions from clinical experience. The antitoxine has great resisting powers against light, heat, etc. Von Ranke first referred to clinical investigations made in December, 1893, with a serum obtained from Behring. Of eight severe cases so treated, seven died, and after death a form of lobular pneumonia was found. Af first it was not quite obvious whether this pneumonia was to be connected with the treatment. In September and October, 1894, Aronson's serum was used. Among nine cases, three died. In one case of severe septic diphtheria, the serum produced no effect. In two severe cases, no change was noted; perhaps the serum was not used in sufficient quantities; one severe and four medium cases recovered without intubation. The author's impressions of this serum were favorable. Von Ranke then tried Behring's serum again. In twelve cases of severe diphtheria there was only one death, and that from the most severe septic form of the disease. The numbers are too small to base conclusions upon, but the investigations will be actively continued. The mortality from diphtheria has hitherto been terribly high. Seitz delt with the preventive inoculation; two cubic centimeters of Behring's serum No. 1 were used for this purpose. The duration of the protection conferred is not long. Septic diphtheria and other complications are not contra-indications to the use of the serum, but such process cannot be expected to be influenced by it. Renal complications, paralysis, and laryngeal stenosis, are rarer with the serum treatment.

